# From coercion to respectful care: women’s interactions with health care providers when planning a VBAC

**DOI:** 10.1186/s12884-022-04407-6

**Published:** 2022-01-27

**Authors:** Hazel Keedle, Virginia Schmied, Elaine Burns, Hannah Grace Dahlen

**Affiliations:** 1 Lecturer of Nursing & Midwifery School of Nursing and Midwifery, Western Sydney University, Locked Bag 1797, 2751 Penrith, NSW Australia; 2 School of Nursing and Midwifery, Western Sydney University, Penrith, Australia

**Keywords:** Vaginal birth after caesarean, VBAC, Midwifery, Continuity of care, Respectful maternity care, Coercion, Birth trauma, Obstetric violence

## Abstract

**Background:**

In many countries caesarean section rates are increasing and this impacts on choices made around mode of birth in subsequent pregnancies. Having a vaginal birth after caesarean (VBAC) can be a safe and empowering experience for women, yet most women have repeat caesareans. High caesarean section rates increase maternal and neonatal morbidity, health costs and burden on hospitals. Women can experience varied support from health care providers when planning a VBAC. The aim of this paper is to explore the nature and impact of the interactions between women planning a VBAC and health care providers from the women’s perspective.

**Methods:**

A national Australian VBAC survey was undertaken in 2019. In total 559 women participated and provided 721 open-ended responses to six questions. Content analysis was used to categorise respondents’ answers to the open-ended questions.

**Results:**

Two main categories were found capturing the positive and negative interactions women had with health care providers. The first main category, ‘Someone in my corner’, included the sub-categories ‘belief in women birthing’, ‘supported my decisions’ and ‘respectful maternity care’. The negative main category ‘Fighting for my birthing rights’ included the sub-categories ‘the odds were against me’, ‘lack of belief in women giving birth’ and ‘coercion’. Negative interactions included the use of coercive comments such as threats and demeaning language. Positive interactions included showing support for VBAC and demonstrating respectful maternity care.

**Conclusions:**

In this study women who planned a VBAC experienced a variety of positive and negative interactions. Individualised care and continuity of care are strategies that support the provision of positive respectful maternity care.

## Background

Women with a previous caesarean can plan a vaginal birth after caesarean (VBAC) or a repeat caesarean before labour. Planning a VBAC can result in having a VBAC or a repeat caesarean before or during labour. Internationally, most women with a previous caesarean have a repeat caesarean yet there is limited data on how many of these women had planned a VBAC. VBAC rates vary between countries with higher rates in some European countries (55% in Finland, 48% in Norway) and lower in the US (13%) and China (10%) [1–3]. In Australia VBAC rates have declined from 13.3% to 2008 to 11% in 2018 [4, 5] and the rates vary between public (18.7%) and private hospitals (5.8%) [6].

There are some identified factors that impact VBAC rates such as having a previous vaginal birth or VBAC, body mass index (BMI), interpregnancy interval, multiple caesareans, and onset of labour [7–10]. There is less research that explores the impact of interactions with health care providers (HCP) on VBAC rates. Lundgren et al. (2019) identified a culture of shared belief in VBAC between HCPs and women in European countries with higher VBAC rates compared to a culture of differing opinions on the value of VBAC in lower VBAC rate countries [11].

Recent qualitative research has identified that women experience varied support from health care providers when planning a VBAC, with many describing negative and unsupportive interactions [11–14]. Effective communication between a healthcare provider and women accessing maternity care is an important domain for providing respectful maternity care [15–17]. Engaging in effective communication includes giving verbal encouragement during labour, providing empathy and listening to women and providing interpreters where required [17].

Interactions with HCP can vary and the type of model of care and the presence or absence of continuity of care can impact this. In Australia, women who have had a previous caesarean have access to a variety of models of care, dependent on location, resources, and availability. In New South Wales (NSW) 12 different models of care have been identified and range from public hospital maternity care, where women will see different healthcare providers at each interaction, to private obstetric and private midwifery models [18]. Data from 2011 found only 8% of women had access to midwifery continuity of care (CoC) models [19], although an increasing number of these models are now becoming available in public hospitals across Australia.

The findings presented in this paper are part of a large sequential mixed methods study into VBAC. The aim of the overall study was to explore women’s experiences of planning a VBAC in Australia. The qualitative phase was a longitudinal study that followed women planning a VBAC throughout their pregnancy and postnatal period. Women recorded their thoughts and feelings following appointment with their health care provider (HCP) using a purpose built smartphone application and all women were interviewed in the postnatal period [20, 21]. The findings of the qualitative study revealed there were four factors that impacted how women felt after planning a VBAC, regardless of birth outcome. The four factors were: having control, having confidence, having a relationship (with health care provider), and having an active labour. These factors influenced whether a woman felt resolved or disappointed after their birthing experience [21].

The qualitative findings were integrated into the next phase of the study which was a national survey. The quantitative results of the experiences of women who had planned a VBAC in the previous five years in different models of maternity care has been published [22]. The aim of this paper is to explore the nature and impact of the interactions between women planning a VBAC and health care providers from women’s perspectives.

## Methods

The data analysed and reported in this paper came from a national Australian survey. The survey was split into two separate pathways, one pathway was for women who were currently pregnant and planning a VBAC at the time of the survey and the second pathway was for women who had planned a VBAC in the past five years and had now given birth. This paper reports on the analysis of the qualitative responses to open ended questions in both pathways of the survey (Table 1.). A content analysis based on the format described by Erlingsson and Brysiewicz [23] was undertaken on the respondents answers to the open ended questions.

### Survey development

The survey development and pilot testing has previously been published [22]. The survey included 114 items that contained demographic, yes/no, Likert scales, open ended questions and validated tools grouped around the four factors. The validated tools included the Mother’s Autonomy in Decision Making (MADM) scale and the Mother’s on Respect Index (MORi) [24, 25]. This paper focuses on the interactions between women and HCPs which were referred to in six open-ended questions in the survey. The questions explored issues around receiving hurtful comments from HCP, positive support from HCP, reasons for hiring a doula, how in control the woman felt and being discouraged from using active labour resources. The list of questions can be found in Table 1.

**Table 1:** Open ended questions

	Question	Number of comments
1	Can you describe any hurtful comments you received?	231
2	Can you describe why you feel/felt your HCP protects/protected you from negativity?	224
3	Describe the positive support	86
4	What were your main reasons for hiring a doula / birth worker?	76
5	Do you have any comments about how in control you felt when planning a VBAC?	70
6	Do you have any comments about being discouraged or prevented from using active labour resources?	34

### Recruitment and data collection

The survey software Qualtrics [26] was used. The survey was distributed through the author’s social media platforms and included an explanatory video, a specific VBAC Facebook page and two paid Facebook / Instagram adverts. The survey was live during the months of March to May 2019.

### Participants

One hundred and seventy-one women responded to the survey about a current planned VBAC and 388 women responded to the survey following a birth where a VBAC had been planned, regardless of birth outcome. In total 559 respondents’ comments were analysed.

### Data Analysis

The qualitative data were downloaded from Qualtrics [26] and sorted in Excel documents. The survey data was then imported into NVIVO 12 [27] for content analysis as described by Erlingsson and Brysiewicz [23]. An inductive method, also referred to as conventional method [28], of content analysis was undertaken due to the lack of research specific to women’s experiences of interactions with HCPs when planning a VBAC in Australia. The content analysis phases of preparation, organisation and reporting were followed [29, 30]. In the preparation phase the units of analysis selected were the answers to the open-ended questions that focused on interactions with HCPs and these answers were read through to get a sense of the data to be coded. The organisation phase followed the process of open coding, creating categories and abstraction [29, 30]. Using NVIVO 12, the answers were coded into initial categories (nodes), the initial categories were then sorted through merging, collapsing, or grouping into hierarchal categories and a codebook was generated. The two datasets from the ‘currently pregnant’ cohort and ‘previous VBAC’ cohort were coded separately and integrated to identify conflicting codes and areas of similarity. No conflicting codes were identified however additional codes were included from the VBAC cohort. The datasets were then merged for the abstraction process.

**Fig. 1:**
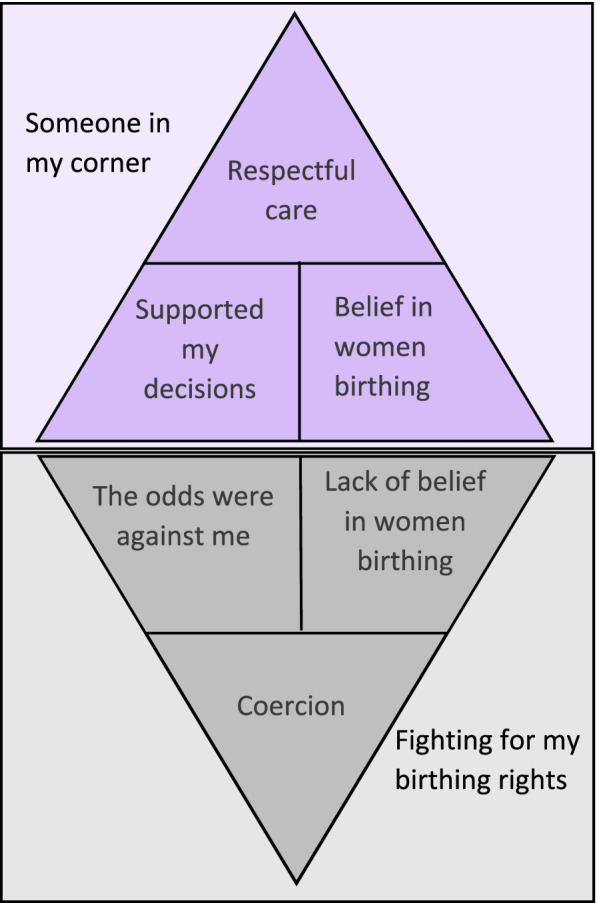
Concept diagram of negative and positive interactions

During the abstraction process the categories were organised into sub-categories and main categories and given titles that were characteristic of the content of the category. Meeting with all authors allowed for refining and organisation of categories and through discussions abstraction continued until there were two identifiable main categories each composed of three subcategories. The hierarchy of categories can be found in Fig. 1.

## Results

### Demographics

In total 559 women answered one or more open-ended questions in either arm of the survey. Most women were aged between 35 and 39 years, stated they were Australian, were married, had a combined income of more than $100,000 and were university educated. Women accessed a range of models of care: 20% had public hospital maternity care which is fragmented in design (seeing different care providers for episodes of care); 16% accessed a private obstetrician; 19% accessed a midwifery group practice (MGP) and 16% of women hired a privately practising midwife (PPM). Women identified 10 different models of care and five women had no health care provider due to a planned freebirth. Of the women who had previously planned a VBAC, 75% of women had a VBAC, 18% had a repeat caesarean during labour and 7% had a scheduled caesarean before labour. Further demographic information can be found in Table 2.

**Table 2:** Demographics of respondents

Demographic	Currently Pregnant n=171 (%)	Given birth in past 5 years n=388 (%)
**Age range**		
18 - 20	0 (0.0%)	1 (0.6%)
21 - 24	5 (2.9%)	24 (6.2%)
25 - 29	47 (27.5%)	100 (25.8%)
30 - 34	67 (39.2%)	159 (41.0%)
35 - 39	48 (28.1%)	90 (23.2%)
40 or over	4 (2.3%)	14 (3.6%)
**Income**		
Less than $40,000	6 (3.5%)	15 (3.9%)
$40,000 - $59,999	15 (8.8%)	33 (8.5%)
$60,000 - $79,999	20 (11.7%)	60 (15.5%)
$80,000 - $99,999	35 (20.5%)	58 (14.9%)
More than $100,000	88 (51.5%)	204 (52.6%)
Prefer not to answer	7 (4.1%)	18 (4.6%)
**Education**		
Less than year 10	1 (0.6%)	3 (0.8%)
Year 10 or school certificate	8 (4.7%)	11 (2.8%)
Year 12 or higher school certificate	16 (9.4%)	49 (12.6%)
TAFE or Diploma	45 (26.3%)	87 (22.4%)
Undergraduate or university qualification	53 (31.0%)	142 (36.6%)
Postgraduate (e.g., graduate diploma, Masters or PhD)	48 (28.1%)	96 (24.7%)
**Ethnicity**		
Australian	126 (73.7%)	307 (79.1%)
Aboriginal and/or Torres Strait Islander	5 (2.9%)	8 (2.1%)
Maori & New Zealander	0 (0.0%)	1 (0.3%)
European	3 (1.8%)	3 (0.8%)
North, Southern and East African and Middle Eastern	3 (1.8%)	6 (1.5%)
South-East & North-East & Southern and Central Asian	13 (7.6%)	28 (7.2%)
North & South American	2 (1.2%)	9 (2.3%)
Other	1 (0.6%)	4 (1.0%)
Missing data	1 (0.6%)	3 (0.8%)
**Relationship**		
Married	120 (70.2%)	305 (78.6%)
Separated	0 (0.0%)	5 (1.3%)
Single	1 (0.6%)	4 (1.0%)
De-facto/ long term relationship	48 (28.1%)	73 (18.8%)
Missing data	2 (1.2%)	1 (0.3%)
**Model of care**		
Public hospital maternity care	41 (24.0%)	71 (18.3%)
Public hospital high-risk maternity care	16 (9.4%)	42 (10.8%)
Shared care (GP and hospital)	18 (10.5%)	21 (5.4%)
General Practitioner obstetrician care	3 (1.8%)	4 (1.0%)
Midwifery Group Practice (CoC with a midwife)	33 (19.3%)	74 (19.1%)
Next birth after caesarean (NBAC) or VBAC clinic	6 (3.5%)	13 (3.4%)
Private obstetrician (specialist) care	24 (14.0%)	67 (17.3%)
Privately practising midwife	20 (11.7%)	67 (17.3%)
Private obstetrician and privately practising midwife joint care	1 (0.6%)	9 (2.3%)
Remote area maternity care	1 (0.6%)	0 (0.0%)
Doula / birth worker	(0.0%)	2 (0.5%)
No health care provider - freebirth	3 (1.8%)	2 (0.5%)
Other	4 (2.3%)	16 (4.1%)
Missing data	1 (0.6%)	0 (0.0%)
**Mode of birth**		
VBAC	-	292 (75.3%)
Scheduled caesarean before labour	-	26 (6.7%)
Repeat caesarean during labour	-	69 (17.8%)
Missing data	-	1 (0.3%)

### Main categories and categories

In total 559 women made 721 comments in response to six questions. Table 1 demonstrates the open-ended questions used in the survey. The questions with the most comments were regarding how the HCP protected the woman from negativity within the health care team (224 comments) and how they responded to or felt about hurtful comments from HCP (231 comments). The open-ended comments in the survey demonstrated the nature and impact of the interactions from the respondent’s (women’s) perspective. The quotes from participants have been identified under the dataset they were, currently pregnant (CP) or previous pregnant (PP), the participant identifying number and their model of care, for example CP6, Public hospital care.

The categories depicting/reflecting negative interactions were grouped under the main category ‘Fighting for my birthing rights’ and the categories that reflected positive interactions grouped under the main category ‘Someone in my corner’, as can be found in Fig. 1. The interactions were on a continuum from negative categories to positive categories. The main category ‘Fighting for my birthing rights’ included the sub-categories: ‘the odds were against me’, ‘lack of belief in women birthing’ and ‘coercion’. The main category ‘Someone in my corner’ included the sub-categories ‘belief in women birthing’, ‘supported my decisions’ and culminates in ‘respectful maternal care’. Further details and definitions of the categories are explored under each of the headings below. Figure 1 shows the continuum of interactions women received from coercion to respectful care.

### Fighting for my birthing rights

Many women who completed the survey experienced negative interactions and lack of support from HCPs. The main category ‘Fighting for my birthing rights’ describes the continual battle women fought, with both HCPs and the system, when seeking a VBAC.

“I stood up for myself and my wants, but it was not easy. I often went home crying after appointments as I felt I was fighting the system” (CP6, Public hospital care).

Analysis of the qualitative comments manifested negativity in three main ways: ‘the odds were against me’, a ‘lack of belief in women birthing’ and ‘coercion’. In the sub-category ‘the odds were against me’, women specified interactions with HCPs that focused on issues that would create difficulty in achieving a VBAC. These issues could be due to characteristics of the woman or resources that were made unavailable or were restricted. The sub-category ‘lack of belief in women giving birth’ focused on the way HCPs dismissed the importance of vaginal birth and demonstrated bias towards repeat caesarean through elevating risks of VBAC and promoting the benefits of caesarean. Finally, the negative interactions reported escalated to the use of many different forms of ‘coercion’, including claims that the woman or baby will die if planning a VBAC and physical assault.

### The odds were against me

In this sub-category women reported comments where the term ‘the odds were against me’ was reflected. The limitations imposed on women included physical characteristics such as body mass index (BMI) and age. Restrictions also related to the use or lack of equipment during labour and the influence of restrictive hospital policies.

The comments from HCPS discouraged women with a higher BMI to plan a VBAC and women were told their BMI could impact their choices during labour, such as access to water immersion; “apparently due to BMI they refused to allow me to use the birthing tub which I had really wanted to as I had a previous 3rd degree tear” (PP358, Public hospital care).

Women also found age impacted how they were treated. From being too young and patronised for not understanding the risks, to being too old -“Comments about my age, over dramatization about risks associated with age, bullying to accept medical intervention, talk about stillbirth that wasn’t relevant and was upsetting” (PP122, Public hospital care ).

Women identified many examples of restrictions that negatively impacted their VBAC experience. The restrictions were predominantly due to staff following VBAC policies and hospital guidelines and/or due to a lack of access to resources such as access to showers and being mobile due to continuous fetal monitoring (CFM) and intravenous cannula’s.

“Cannula restricted some movement, but it was more that the hospital midwives didn’t really provide me with advice on different positions to help” (PP331, Public hospital care).

Many women who wished to use water immersion during labour and/or birth were disappointed when they were unable to access it due to lack of resources, policy or due to the combined requirement of CFM - “Was not allowed water-based relief due to constant need for baby heart monitoring” (CP2, Public hospital care).

A lack of access to water immersion was one of the reasons some women chose to birth outside of the hospital, either with a PPM or to freebirth.

“One of the reasons that I chose to home birth was BC [Birth Centre] at our local hospital did not allow VBAC mamas to use the birth pool and insisted on CTG monitoring, which meant that I could not have an active labour” (PP196, PPM care).

### Lack of belief in women giving birth

Women in the survey were able to describe interactions that highlighted the potential bias of the HCPs towards caesareans and a lack of belief in VBAC as a viable or suitable option for women. This bias was demonstrated through questioning the validity of wanting a VBAC and a belief that the woman’s body was at fault.

Some women found HCPs didn’t understand the desire to experience a vaginal birth and were flippant in their comments about VBAC - “I was questioned why I would possibly want to have a natural birth” (P196, Public hospital care).

Bias towards caesareans could be displayed in comments favouring the ease of organising a scheduled caesarean for the staff, “Dr telling me that she prefers cs as easier to plan and safer” (CP34, Public hospital care) and “basically told it’s just the best to book a c-section and not go through all the hassle and stress of a VBAC” (CP59, Public hospital care).

The lack of belief in the importance of vaginal birth extended to comments regarding the disbelief in the woman’s body to be able to have a VBAC. Language that questioned the capacity of women to birth was used, sometimes during internal examinations “Oh your body won’t do this on its own. I can’t even reach your cervix” (PP77, Public hospital care) and “Not enough space here” (PP78, MGP care).

Comments from HCPs used language that put the woman and her baby in opposition to each other, suggesting an inability of the woman’s body to grow a baby capable of being birthed vaginally.

“Surgeon said there were no contraindications to me trying for a VBAC but that I probably had CPD [Cephalopelvic Disproportion] so she wouldn’t recommend a VBAC. Comments about my stature and size of my baby, despite being of average height and baby being above average but not macrosomic” (CP166, MGP care).

The effect of the continued negative language about women’s bodies offended women.

“Resources wasted on me, time waster, accept my body won’t birth, I’m also sick to death of terminology like unfavourable cervix, failure to progress, overactive uterus like I’m deformed and broken. I just don’t like being prodded and pricked like a science experiment! I don’t want to birth in that atmosphere and I’m unique. Not average!” (PP370, Public hospital care).

### Coercion

Women described coercive comments in the question regarding control when planning a VBAC and when describing hurtful comments. Most women experienced hurtful comments from a HCP at some point during their VBAC journey. Coercion escalated from exaggerated facts to HCPs stating the woman, or more commonly the baby, will die if she continues with her decisions and choices.

Women found information from HCPs regarding VBAC and repeat caesarean was unbalanced and favoured repeat caesarean.

“One Ob kept emphasising VBAC risks and pretty much stated false facts when I mentioned risks of c-sections, particularly risks of several c-sections if I want more children. I decided to keep my opinions to myself in this appointment after this as I saw no point in expressing them anymore” (CP1, MGP care).

Incorrect facts were often mixed with opinion and expressed in alarming language, which gave confusing and unsupportive information to women.

“A doctor at the back up hospital told me that I was at considerable risk of uterine rupture and that my previous vaginal birth and VBAC were no indication that I had a higher chance of successful VBAC this time. She said that I was probably at more risk because I had had a large baby during my last VBAC and therefore my scar would have been made weaker by accommodating a large baby” (PP133, PPM care).

Some women were unaware that the comments they received were likely biased towards a repeat caesarean. A woman who had a private obstetrician had an antenatal appointment at term and found herself no longer supported to wait for labour to occur.

“I didn’t go into labour. Had an internal examination at 40+2 and was not dilated so had to have a caesarean” (PP187, Private Obstetrician care).

Some women experienced subtle behaviours from HCPs that resulted in women being silenced through being made to feel childish or over emotional.

“When I tried to be assertive and show the medical staff I had done research and was making informed decisions, they spoke over the top of me or told me I was emotional” (PP14, Normal Birth After Caesarean clinic).

During labour and birth one woman found she was discouraged through threatening language from using her voice during contractions; “I was told that by making noise, I was depriving my baby of oxygen” (PP127, Public hospital care).

Language often escalated to be degrading such as suggesting a VBAC would stretch the vagina and the implications on future sexual intercourse. Some participants found comments were redirected to partners or male support people in order that they have a part in changing the woman’s mind.

“I was told ‘you are stubborn and ignorant, and I can say that because I’m the doctor’ and she then told my partner’s friend whom she assumed was my brother because we called him uncle to ‘talk some sense into your sister’ she also said ‘aww your poor belly, look at your skin, don’t worry it will go back,’ even though I like my skin” (PP20, Public hospital care).

Women who had CoC with a midwife often had to attend at least one antenatal clinic with a doctor and this could be a time where they received hurtful comments. One woman who was planning a VBAC at home described her experience.

“In regards to my IM [Independent Midwife] I felt in control. With the one hospital Dr appointment I had I felt belittled, pressured, bullied and discriminated” (PP230, PPM care).

Coercion was used to change women’s behaviour, either regarding their plan to have a VBAC or the choices they make around planning a VBAC. A woman who was initially planning a VBAC in hospital described how coercion and compliance was introduced by a HCP during a NBAC (Next Birth After Caesarean) class.

“I switched to private midwifery care from hospital care due to my desire to be supported and have control over my birth. The hospital experience was completely the opposite of the quality care I received from my midwife… The last straw for me was attending the local public hospital NBAC class where the medical professional said, I quote “The only times I have seen things go wrong is when women don’t obey the rules”. I left the class horrified and saying to my Doula who also attended the class with me “I’m going to be one of those women who don’t ‘obey’ the rules”. I then sought out and found the most amazing midwife who supported me to achieve my VBAC and first homebirth experience. Hands down the gentlest most empowering birth experience I have ever had” (PP46, PPM care).

One doctor sought to deter a woman from planning a VBAC by threatening that if anything went wrong it would have a traumatising impact on staff ; “Ob [obstetrician] told me her staff would be traumatised if something went wrong during my VBAC” (PP110, Public hospital care).

Verbal threats could escalate to potentially devastating actions. One woman stated the actions of HCPs in the hospital that negatively impacted the woman and her family.

“My VBAC attempt was not supported by the hospital… They reported me to Child Protective Services for not booking an elective c-section (no medical reason to book one) so on the day of the labour I felt I didn’t have any power” (PP14, NBAC clinic care).

Many women experienced comments that threatened the death of themselves or their baby due to planning a VBAC, often described by the women as being given the “dead baby card” (PP128, Public hospital care).

“One obstetrician I saw at 20 weeks said “if you try for a VBAC, your husband will end up with a dead wife, a dead baby & a toddler to raise on his own”. I have obviously refused to see him again & have booked all future hospital appointments with a VBAC supportive Ob” (CP161, Public hospital care).

Coercion could result in assault with procedures used without asking, use of physical force and forced procedures. Women in the study experienced obstetric violence that was extreme, “I was physically man handled onto the bed” (PP370, Public hospital care), to receiving unconsented procedures such as episiotomies or instrumental births.

### Someone in my corner

Moving along the interaction continuum are the positive interactions under the main category ‘Someone in my corner’. This main category identifies the overall sense from women that their doula or HCP were supporting, advocating, and facilitating their desire to plan a VBAC.

The categories are HCPs ‘believing in women birthing’, HCP / doula ‘being an advocate’ for the woman and culminating in the woman receiving safe, ‘respectful maternity care’. The sub-category ‘belief in women birthing’ displays comments were women felt the HCP believed in the woman’s ability to have a VBAC. The sub-category ‘Supported my decisions’ highlighted occasions where HCPs showed they supported the woman’s wishes and choices and interactions where the woman felt the HCP/doula would be an advocate for her choices. In the final positive interaction sub-category, ‘respectful maternity care’, comments regarding CoC and respectful relationships with HCPs are presented.

### Belief in women birthing

The questions ‘Can you describe why you feel/felt your HCP protects/protected you from negativity?’, ‘Describe the positive support’ and ‘Do you have any comments about how in control you felt when planning a VBAC?’ provided answers on positive interactions with HCP. Women described these interactions as the HCP believing in VBAC as an option and encouraging women along their VBAC journey.

“My MGP midwife is pro-VBAC and very encouraging. She hasn’t had anything negative to say and this is so reassuring” (CP120, MGP care).

Women found encouragement from a variety of HCPs, a woman who had shared care with her GP found consistent support across the HCPs she encountered.

“I felt quite informed about VBAC prior to planning one and am quite health literate. I sought supportive care from a known GP who would be supportive of my choice. Shared care with this female doctor was wonderful and she was fully respectful and encouraging of my decisions. The midwives I saw at the hospital were also encouraging. I had mixed interactions with obstetricians at the hospital appointments. One was extremely encouraging, the other not so” (PP264, GP shared care).

Some interactions with HCPs made women feel that VBAC was an uncommon choice. A woman who didn’t have access to CoC in her local area found encouragement from some midwives which escalated after her VBAC.

“Mostly, midwives were encouraging of me “trying for a VBAC”, however it was as though it was a rare occurrence! Some very kind midwives contacted me after my birth and congratulated me on achieving my VBAC!! It was absolutely incredible encouragement” (PP364, Public hospital care).

Alongside encouragement, some women benefited from continued positive reinforcement using “positive language, empowering me the whole way” (PP79, MGP care), which provided support when women were doubting their VBAC decision.

Midwives promoted the use of VBAC literature, websites and social media support groups for women to gain knowledge and hear positive VBAC stories.

“All positive reinforcement and encouragement. Literature given and social media- based support groups were suggested” (CP120, MGP care).

The feeling that the HCP believed in their ability to have a VBAC sometimes manifested itself in the flexibility around hospital guidelines and policies. Women found HCPs who took an individualistic approach to suggested guidelines and policies were more supportive about VBAC.

“I was able to proceed how I wanted in spite of it being against their policies. The language to communicate about policy was framed in a way that indicated that I needed to be aware of it, but at the end of the day I was free to choose” (PP120, MGP care).

Women benefited from HCPs who consistently believed in their ability to have a VBAC, regardless of actual mode of birth. A woman who was a midwife herself and planning a VBAC after two caesareans (VBA2C) chose a specific private obstetrician due to personal experience. She described her experience of positive support and belief in VBAC.

“Very encouraging, I was in a hospital where VBA2C occurred at semi regular intervals. My ob and I both supported a woman through a VBA2C which is why I chose him as an Ob because of how he treated her. He used evidence to guide his recommendations to me, never used scare tactics. Was honest, believed in me and even when I had another cesarean, he allowed me to have a maternal assisted CS and asked when my VBA3C was going to be” (PP 288, Private obstetrician care).

### Supported my decisions

The sub-category ‘Supported my decisions’ captured comments regarding positive support, advocacy, protecting from negativity and reasons for hiring a doula. Women identified advocacy as an important aspect of why they felt their HCP protected them from negativity. This sub-category was demonstrated through HCPs supporting women’s choices and wishes and in giving women the confidence to self-advocate.

Women frequently highlighted the advocacy actions of midwives. One woman had her PPM attend appointments. “My midwife attended any external antenatal appointments I had with the hospital obstetrician often explaining anything I was unsure of. She was amazing, clarification and advocate for my choices” (PP40, PPM care). Another woman felt that having a PPM with her in the hospital subtly advocated for her as she states, “I feel like the hospital knew they couldn’t force anything on me because of my midwife’s presence” (PP250, PPM care).

Women who had care through MGP also highlighted how their midwives advocated for them.

“They advocate for us and what we are trying to achieve. They protect us from a lot of the negative comments that come through from other health professionals” (CP32, MGP care).

MGP midwives were identified as speaking up for the woman’s wishes with the multidisciplinary team, “My MW bore a lot of the brunt from the OB and went into bat for me multiple times” (PP103, MGP care). This was also the case when navigating hospital systems.

“I was respected and treated as a consenting adult with my midwife. I was able to actually have informed conversations and figure out the best options for my care. With my OB appointments during pregnancy I had to fight for even a shred of dignity and respect. The fact that I was comfortable, and my midwife was able to help me navigate the rest of the hospital meant I felt protected by her” (PP108, MGP care).

Advocacy was experienced during labour, especially when health professionals had differing opinions. Women who had CoC with a midwife found their midwife sheltered them from interventions and actions from other HCP; “She had my back when the Dr on duty in birth suite suggested rupturing my membranes. I had only just arrived at hospital and was already 8 cm” (PP157, PPM care).

“Allocated MGP [midwife] was like the bodyguard for the short time I was in labour. She met me outside the hospital, escorted me into my room (no check-ins or other interruptions) and then instructed staff that no one was to enter unless she specifically asked them to” (PP386, MGP care).

Some women who had CoC with a private obstetrician described how the doctor had been their advocate by influencing staffing or decisions on care “he advocated for me and even requested a change of midwife during my labour to someone who was more aligned to my desires” (PP98, Private Obstetrician care).

Some women responding to the survey hired doulas regardless of the model of care they experienced. Doulas were seen by women as advocates, someone employed to support and speak out when the woman was in labour; “to have someone to advocate for me if I could not and to help me to make my wishes to come true” (PP131, MGP care).

“To be on my side and to be a source of strength during the birth if the OB started to unnecessarily pressure me into things I didn’t want to do” (PP57, Public hospital care).

A woman who had planned a HBAC (homebirth after caesarean) with a PPM also hired a doula to provide extra support and extra advocacy:

“Wanted extra support by someone with experience. Should I need a hospital transfer, I wanted many advocates” (PP300, PPM care).

### Respectful Maternity Care

Although there were fewer positive comments from women who accessed public hospital, fragmented care, some women found support and “Encouragement toward the VBAC” (CP44, public hospital care) from their interactions with midwives and doctors.

“Was given information and have been supported by both midwives and doctors that I could try for a VBAC” (CP41, public hospital care).

“I felt respected by both the midwife and the O&G in my decision” (PP17, public hospital care).

There were more positive comments that indicated respectful maternity care from women who had CoC and many women in this study actively sought CoC when planning a VBAC. Continuity was accessed with private or publicly funded midwives or through private obstetricians. Women identified the impact CoC had on feeling in control when planning a VBAC. A woman who had antenatal care in a high-risk clinic identified the lack of CoC as contributing to fear.

“Without continuity of care I had a different doctor each visit giving their opinion on whether I could/couldn’t have a VBAC. It was extremely frustrating and actually added uncertainty and fear to my pregnancy and birth” (PP130, High risk clinic care).

In comparison, a woman who had CoC with MGP found: “continuity of care meant that even when I wasn’t able to be in control, my decisions were still respected” (PP251, MGP care).

Receiving CoC protected women from having to retell and relive previous traumatic experiences. A woman who had a PPM and had planned a HBAC but “in the end I needed to be induced at hospital due to PROM and GBS+” (PP124, PPM care) found solace in continuity when things did not go to plan.

“My private midwives were amazing. Through continuity of care they didn’t just know my story, but also how important it was that my experience was different this time around. I also had a number of hospital midwives approach me during my time in hospital apologizing for my previous experience and telling me that they’re frustrated that the public health system doesn’t support women properly” (PP124, PPM care).

Many women also accessed continuity through hiring a doula either when there was no continuity available with a health care professional or in addition to having continuity with a health care professional. A woman who had a private obstetrician hired a doula to focus on labour care.

“Continuity of care from someone who would be present for the whole of my labour. I had continuity of care from my obstetrician, but I knew he would not be there providing midwifery support for the whole of my labour” (PP340, Private obstetrician care).

A woman who had CoC through MGP hired a doula to ensure continuity in case others couldn’t be there; “My partner was working away until my due date & I was afraid he’d miss the birth, so I wanted a backup plan… I was also very mindful my confidence in my MGP Midwifery was invaluable and if she couldn’t be there I needed support from another source” (PP144, MGP care).

 Some women were aware of the impact of choosing care outside of recommended guidelines and found protection through having CoC.

“I was in control of my decision, but clearly understood that the decision I was making was not in line with the recommendations made by any of the doctors involved in my care. My midwife gave me all the control I needed to counter this ‘disapproval’ from the doctors within the public health system” (PP156, PPM care).

Positive interactions with an HCP were made up of three categories: ‘belief in VBAC’, ‘Supported my decisions’, and ‘respectful maternity care’. Women wanted HCPs who would advocate for them within the hospital system and often hired doulas to help them self-advocate when required. Women sought CoC to develop a relationship based on respect and knowledge of past experiences and current choices. Finally, women benefited from HCPs who believed in VBAC and who offered positive reinforcement and an individualistic approach to policies and guidelines.

## Discussion

The findings show that the nature of HCP and woman interactions were on a continuum from respectful maternity care to coercion. This discussion will explore respectful maternity care and coercion and then explore the reasonings behind these interactions, considering why they occur and what strategies can be used to prevent negative interactions and increase positive interactions.

### Coercion

When analysing women’s words we found that coercion was at one end of the continuum. Definitions of coercion commonly focus on the use of power or fear to gain compliance. Reproductive coercion has been defined as behaviours that interfere with the autonomous reproductive decision-making of women [31]. Kotaska [32] identifies that coercion can take different forms within the HCP-woman relationship and can include giving false or exaggerated facts to influence options, withholding risk information of a recommended treatment, demeaning women, reporting to child protection services due to the woman’s decisions and threatening to withdraw care. In this study, the open-ended responses from women included 231 hurtful comments that reflected coercive interactions that included making threatening comments to women regarding the death of their baby or themselves if they (women) continued planning a VBAC. A study from the US found a third of doula’s and nurses witnessed care providers threatening women that their baby might die in labour and delivery wards if the woman did not agree with a suggested intervention [33]. A content analysis on responses in the Listening to Mothers in California survey found both positive and negative interactions with HCPs, the negative interactions included HCPs who were rude, judgmental and rushed [34]. A Canadian study found participants who declined aspects of maternity care experienced coercive and pressured interactions which could result in the individual losing trust in the health care provider [35]. Using threatening and hurtful comments and behaviours can be categorised as non-dignified care in the ‘disrespect and abuse in childbirth’ definition [36] which is also described as obstetric violence [37].

Coercive comments and behaviours from HCP can negatively impact women who have a history of trauma and can cause re-traumatisation [38–40]. Many women who access maternity care have experienced traumatic events which can include their previous caesarean experience. In the VBAC in Australia survey 69% of women stated their previous caesarean was a traumatic experience [22]. A meta-ethnography on birth trauma found women experienced overwhelming negative emotions during their subsequent pregnancy [41]. Women with a previous traumatic experience who then have an exposure to a subsequent traumatic experience, such as coercion from a HCP, have a higher probability of developing PTSD [42, 43].

### Conflicting information

In this study women experienced conflicting recommendations from medical and midwifery professionals, particularly when women received midwifery CoC. Women who had CoC with a midwife were assigned at least one antenatal appointment with a doctor. They may have also had interactions with doctors during labour and birth and/or in the postnatal period. It was often during these interactions women would experience negative and hurtful interactions which were contradictory to the care they were receiving from their CoC midwife. Cummins et al. [34] conducted focus groups with consumers and midwives to explore the qualities of midwifery CoC and found there were times of disagreement between obstetricians and midwives. Women were aware of the tension, which led to confusion and confliction. Disagreements are often based on divergent philosophies with midwives supporting physiological birth and obstetricians favouring interventions which impact on women who are seeking a physiological and active labour and birth, including access to water immersion [44, 45].

### Unconscious bias

Exploring the role of unconscious / implicit bias in the HCP can potentially explain the behaviours and attitudes exposed in interactions reported in this study. Unconscious biases are: learned and unsupported views that favour one thing, person or group over another, which quickly impact decision making [46]. Research suggests HCPs exhibit similar levels of implicit bias as the wider population [47]. In healthcare this bias can be found in direct care and health outcomes, in the provision of maternity care organisations, HCP education and training and within research [46, 48]. Dejoy et al. [49] found women with higher BMI’s experienced at least one stigmatising interaction and suggested weight bias from HCPs is a contribution to these negative interactions. Women from diverse cultural backgrounds also experience similar problems. Researchers have found that racial bias impacts mode of birth decision making [50]. Interviews with obstetricians regarding caring for non-English speaking migrants of Micronesia in Hawaii found biased attitudes due to language and stereotypical expectations. One obstetrician explained that the consenting process for a repeat caesarean was easier than explaining and consenting to a VBAC, suggesting a communication and caesarean bias [50]. In this study women identified that having a higher BMI or their age (too young / too old) and planning a VBAC in particular led to more negative interactions with HCPs.

### Respectful Maternity Care

When analysing women’s words in this study we found that respectful maternity care was at the other end of the continuum. Respectful maternity care is defined by the World Health Organization as “care organized for and provided to all women in a manner that maintains their dignity, privacy and confidentiality, ensures freedom from harm and mistreatment, and enables informed choice and continuous support during labor and birth” [51]. Following a qualitative synthesis, Shakibazadeh et al. [17] provided twelve domains of respectful maternity care which include engaging in effective communication, respecting women’s choices that strengthen their capabilities to give birth, providing equitable maternity care and CoC. In this study, accessing CoC contributed to positive interactions encompassed in respectful maternity care and resulted in women feeling in control of their decisions and outcomes through relationships based on trust and equity. Our previous published findings revealed that women who had CoC with a midwife were happier with their CoC experience, reported more positive support from their HCP and had longer appointments compared to standard care, or CoC with a doctor [21, 22].

There are many known benefits of midwifery CoC, such as reducing preterm birth rates and decreasing interventions during labour and birth [52]. The quantitative results of this survey found CoC midwives spent more time with women during appointments compared to CoC with a doctor or standard antenatal care [22]. A study exploring midwives’ perceptions of respectful maternity care in Iran highlighted the importance of communication and relationship, providing safe and ethical care and safeguarding the dignity of women [53] and Hildingsson et al. [54] found women in Sweden who had a known midwife during labour and birth had more positive birth experiences. A study from the USA found women planning a VBAC had lower intervention rates with certified nurse-midwives (CNM) compared to obstetric care [55]. In Australia, Cummins et al. [34] found midwifery CoC also impacted the traditional hierarchical model of maternity care to one based on equality and respect. The quantitative data from this survey also showed where women had midwifery CoC they had higher scores on the Mothers on Respect Index (MORi) and Mothers on Decision Making (MADM) index [22].

### Strategies to increase respectful interactions

Introducing an obstetric violence framework into national law can be a powerful tool for addressing cases of coercion and body autonomy [56]. Obstetric violence was recognised by Venezuelan law in 2007 and Latin American countries have since implemented obstetric violence legislation. Recognition of this law has provided avenues for women to take legal action against health professionals and organisations and provided a framework to identify systemic failures that manifest in disrespectful maternity care [37, 56, 57]. An obstetric violence legal framework isn’t currently available in Australia and women who attempt to seek legal recompense struggle within the criminal and civil justice system due to economic, racial and gendered discriminations [58]. In this study women described experiences of coercion that ranged from hurtful comments to physical restraint and non-consented procedures. A legal framework for obstetric violence alongside public education on the definition, impact and legal avenues for women who experience obstetric violence may act as both a deterrent and basis for recompense for women who receive coercion and abuse in maternity care [56].

There should be a focus in clinical practice and research on strategies to decrease negative interactions and increase positive interactions. Strategies to decrease the use of coercion and obstetric violence should recognise the broader societal impact of violence against women [37]. Strategies may include HCP training on the impact of bias, race and culturally competent care, and individualised care [37, 59]. Implementing trauma-informed maternity practices and training can promote positive interactions based on respect, empowerment and collaboration [38]. A qualitative study from the USA on women who had experienced multiple lifetime traumatic exposures highlighted the importance of developing trusting relationships with empathetic HCP as an opportunity to disclose trauma and to have positive maternity experiences [60]. Midwifery models of continuity of care need to be expanded to include women with a previous caesarean and be made available to more women across Australia.

### Limitations of the study

The limitations of the national survey have been described in a previous published paper [22] and include the recruitment of the survey using a self-selected sample of women accessed through social media. The respondents of the survey were English speaking and were predominantly born in Australia, with around 2% identifying as Aboriginal and/or Torres Strait Islander. A large proportion of women were university educated with higher incomes which indicates a well-educated and well-resourced population. Further research is required to ascertain the views of the wider Australian community including women from culturally and linguistically diverse communities, women from lower socio-economic communities and women who identify as Aboriginal and/or Torres Strait Islander.

## Conclusions

In this study women who planned a VBAC experienced a variety of positive and negative interactions. Negative interactions included the use of coercive comments such as threats and demeaning content. Positive interactions included supporting VBAC and examples of respectful maternity care. Individualised care and continuity of care are strategies that support the provision of positive interactions and respectful maternity care. A framework for obstetric violence alongside public education on the meaning of this term could be introduced in Australia to try and reduce these negative experiences for women.

## Data Availability

The data that support the findings of this study are available on request from the corresponding author HK. The data are not publicly available due to them containing information that could compromise research participant privacy/consent.

## Abbreviations

CoC: Continuity of Care
GP: General Practitioner
HCP: Health Care Provider
MGP: Midwifery group practice
PPM: Privately Practicing Midwife
VBAC: Vaginal Birth after Caesarean
